# Tick species, tick-borne pathogen distribution and risk factor analysis in border areas of China, Russia and North Korea

**DOI:** 10.3389/fvets.2025.1529253

**Published:** 2025-02-11

**Authors:** Pengfei Min, Jianchen Song, Shaowei Zhao, Zhen Ma, Yinbiao Meng, Zeyu Tang, Zhenyu Wang, Sicheng Lin, Fanglin Zhao, Meng Liu, Longsheng Wang, Lijun Jia

**Affiliations:** ^1^Engineering Research Center of North-East Cold Region Beef Cattle Science and Technology Innovation, Ministry of Education, Yanbian University, Yanji, China; ^2^State Key Laboratory for Diagnosis and Treatment of Severe Zoonotic Infectious Diseases, Key Laboratory for Zoonosis Research of the Ministry of Education, College of Veterinary Medicine, Jilin University, Changchun, China

**Keywords:** tick species, tick-borne diseases, risk factors, border areas of China, tick-borne pathogen distribution

## Abstract

**Introduction:**

Ticks are important ectoparasites of livestock. Ticks and tick-borne diseases (TBDs) cause losses to the animal husbandry industry and also present a major hidden danger to public health and safety. However, the tick species and prevalence of TBDs in border regions of China, Russia, and North Korea remain unknown. The purpose of this study was to identify the tick species and tick-borne pathogens endemic in these regions.

**Methods:**

Morphological and molecular identification of ticks was performed by microscopy and polymerase chain reaction (PCR), and the distribution of tick species, pathogen, and risk factors of infection were analyzed.

**Results:**

In total, 1,187 ticks were collected from the border areas of 13 localities in eight cities. Five tick species were identified: *Haemaphysalis longicornis* (39.68%), *Ixodes persulcatus* (25.36%), *Haemaphysalis japonica* (15.50%), *Dermacentor silvarum* (15.42%), and *Haemaphysalis concinna* (4.04%). There were more female than male ticks, and nymphs were the least frequently collected. *I. persulcatus* was the main species in the forest environment, while *H. longicornis* was the main species in grasslands and animal surface. Four pathogens were detected: *Rickettsia*, *Bartonella*, *Anaplasma*, and *Babesia*.

**Discussion:**

Pathogen detection in ticks differed significantly among the environments and between Sexes. There were significant differences in the proportion of ticks infected with *Rickettsia*, *Bartonella*, *Anaplasma*, and *Babesia* among regions, species, sexes, and environments. The results of this survey of the tick species in border areas of China, Russia, and North Korea provided a scientific basis for the prevention and control of TBDs.

## 1 Introduction

Ticks are important ectoparasites of livestock and can be divided into three families: Ixodidae, Argasidae, and Nuttalliellidae (1). Although most common tick species are distributed throughout various provinces and cities in China, some are unique to a certain region, which may be related to environmental differences (2). Currently, 907 species of ticks have been reported worldwide. About 120 tick species have been identified in China alone, with most (80%) being hard ticks (3). Under normal circumstances, the body length of ticks is approximately 2–15 mm, although body size can rapidly increase while sucking blood (4). Tick development occurs as a process of incomplete metamorphosis, which can be divided into the egg, larva, nymph, and adult stages (5). Ticks can be classified based on the number of hosts and molting sites (6). In addition, ticks can readily adapt to harsh environmental conditions and some species can survive without food for long periods (7). Tick distribution is closely related to climate, soil, water, geographical environment, hosts, and other factors (8, 9). Ticks and tick-borne diseases (TBDs) not only cause harm to the animal husbandry industry, but also pose a major hidden threat to public safety and health (10). Moreover, many tick species can cause anemia and other diseases, as well as transmit various pathogens to the hosts, including *Anaplasma, Bartonella, Rickettsia, Babesia*, and Tick-borne encephalitis virus (11).

*Rickettsia* are small Gram-negative bacteria with an obligate intracellular life cycle circulating between mammalian hosts and hematophagous arthropod vectors in nature. *Rickettsia* are transmitted to mammalian hosts during blood feeding by infected ticks and mites (12). *Rickettsia* are categorized as belonging to the spotted fever group (SFG), typhus group (TG), transitional group (TRG), and ancestral group (AG) (13). The main clinical symptoms of *Rickettsia* infection in humans are fever, headache and nausea. Severe patients may die. Overall, the public health burden of tick-borne Rickettsioses remains significantly underestimated (14). *Anaplasma* belongs to the family *Anaplasmataceae* of order *Rickettsiales*. The genus *Anaplasma* includes *Anaplasma marginale*, *Anaplasma centrale*, *Anaplasma bovis*, *Anaplasma ovis*, and *Anaplasma phagocytophilum* transmitted by ticks. Different types of *Anaplasma* cause different clinical symptoms. *A. phagocytophilum* mainly cause fever, abortion, and decreased milk production (15). *A. marginale* has the most severe symptoms and may lead to death of livestock if not treated in time (16). *A. centrale* is the least pathogenic and is often used in vaccines (17). Today, *Anaplasma* still has effects on human and animal health at the global level. *Bartonella* species are gram-negative, and zoonotic bacteria belonging to the α2-subgroup of proteobacteria (18). It is spread to mammals mainly by blood-sucking arthropods. Cat scratch disease (CDS) is the most harmful disease to humans caused by *Bartonella*, with approximately 12,000 cases reported annually in the United States (19). Although the incidence is not high, we still need to take it seriously. *Babesia* is a protozoan parasite of the phylum Apicomplexa. Babesiosis is a worldwide tick-borne zoonosis caused by hemoprotozoan parasites of the genus *Babesia* (20). Ixodes ticks are the main vectors of *Babesia* spp. Clinical manifestations of Babesiosis are mainly related to the immune function of the host. *Babesia bovis* can have a serious impact on the livestock industry. The economic loss to China is up to 60 million dollars per year (21). Therefore, scientific prevention and control of *Babesia* is crucial for the livestock industry.

In terms of incidence, TBDs are the most serious vector-borne diseases in the animal husbandry and veterinary fields, and the second most common human vector-borne diseases after mosquito-borne diseases (22). Tick species and the prevalence of TBDs in border areas of China, Russia, and North Korea remain unknown. Therefore, the aim of this study was to identify the tick species and pathogens in border areas of China, Russia and North Korea, and to analyze potential risk factors, so as to provide a scientific basis for the prevention and control of TBDs.

## 2 Materials and methods

### 2.1 Collection of tick samples

Free ticks were collected using the cloth flag method. The collection sites consisted of grasslands and forests with lush vegetation close to a water source. When sampling, the gauze was laid flat on the grass and moved slowly by hand with a stick. At regular intervals, a magnifying glass and tweezers were used to transfer ticks from the gauze to a 15 mL centrifuge tube and relative information was recorded. Farms and villages were randomly selected. After obtaining the consent of farmers, the surfaces of livestock (cattle and sheep) were checked for the presence of ticks at the preferred attachment sites, such as behind the ear, perineum, and lower abdomen. During collection, the head of the tick was clamped with elbow tweezers and the mouthparts were gently rotated and pulled out perpendicular to the body surface. The samples were then placed into a labeled plain 15 mL centrifuge tube.

### 2.2 Morphological identification of ticks

Adult ticks with relatively complete morphology of different species were selected and washed three times with sterile water to remove dust from the surface of the tick and soaked in phosphate-buffered saline (23). The morphological structures of different species of male and female ticks were observed with a stereomicroscope. Images were captured and stored following appropriate taxonomical keys (24).

### 2.3 Tick DNA extraction

All ticks were used to extract DNA, and each tick was tagged individually. The ticks were ground to powder in liquid nitrogen. Tick DNA was extracted using a tissue Genomic DNA Extraction Kit (Tiangen Biotech (Beijing) Co., Ltd., Beijing, China), in accordance with the manufacturer’s instructions, and stored at-20°C until further use.

### 2.4 Detection of tick-borne pathogens

Species-specific primers were used to amplify the 16S ribosomal DNA (*16SrDNA*) gene of tick (45), outer membrane protein-A (*ompA*) gene of *Rickettsia* (41), citrate synthase (*gltA*) gene of *Bartonella* (42), 16S ribosomal RNA (*16SrRNA*) gene of *Anaplasma* (40), chaperonin-containing t-complex polypeptide 1 (*CCTeta*) gene of *Babesia* (37), and major piroplasm surface protein (*MPSP*) gene of *Theileria sinensis* and *Theileria orientalis* (43, 44). The primers used in this study are listed in Table 1. The PCR reaction was conducted with a 25-μL reaction volume comprising 1 μL of each primer (10 pmol), 3 μL of template DNA (50–60 ng/μl), 2 μL of deoxynucleotide triphosphates (Takara Biotechnology (Dalian) Co., Ltd., Dalian, China), 2.5 μL of 10× Ex *Taq* buffer, 0.25 μL of Ex *Taq*, and 15.25 μL of distilled water. The PCR reaction conditions are presented in Table 2.

**Table 1:** PCR primers for ticks and pathogens.

Pathogen	The name of the gene	Primer sequences (5′-3′)	Fragment size (bp)
*Anaplasma*	*16SrRNA*	F-TACCTCTGTGTTGTAGCTAACGC R-CTTGCGACATTGCAACCTATTGT	426 (40)
*Rickettsia*	*ompA*	F-ATGGCGAATATTTCTCCAAAA R-AGTGCAGCATTCGCTCCCCCT	530 (41)
*Bartonella*	*gltA*	F-GGGGACCAGCTCATGGTGG R-AATGCAAAAAGAACAGTAAACA	356 (42)
*Babesia*	*CCTeta*	F-GCCCGCAGGTCATCATAAAGT R-CATTTTGTGCCAGCGTTTTG	1,008 (37)
*T. sinensis*	*MPSP*	F-CACTGCTATGTTGTCCAAGAGATATT R-AATGCGCCTAAAGATAGTAGAAAAC	887 (43)
*T. orientalis*	*MPSP*	F-CTTTGCCTAGGATACTTCCT R-ACGGCAAGTGGTGAGAACT	776 (44)
Tick	*16SrDNA*	F-CTGCTCAATGATTTTTTAAATTGGGTGG R-CCGGTCTGAACTCAGATCAAGT	460 (45)

**Table 2:** Reaction conditions of various primers.

Primer name	Pre-denaturation temperature(°C)/time(s)	Denaturation temperature(°C)/time(s)	Annealing temperature(°C)/time(s)	Stretching temperature(°C)/time(s)	Temperature of reextension(°C)/time(s)	cycle	Storage temperature(°C)
*Anaplasma*	94/300	94/15	55/35	72/55	72/480	40	4
*Rickettsia*	95/300	95/30	50/30	72/30	72/480	35	4
*Bartonella*	94/300	94/30	55/30	72/30	72/420	35	4
*Babesia*	95/300	95/30	51.4/30	72/30	72/480	35	4
*T. sinensis*	94/300	94/60	56/60	72/60	72/420	35	4
*T. orientalis*	94/300	94/60	58/60	72/60	72/420	35	4
Tick	95/300	95/30	54/30	72/50	72/420	35	4

### 2.5 Sequencing and phylogenetic analyses

All samples verified as positive by agarose gel electrophoresis were sent to Shanghai Shenggong Biotechnology Company for Sanger sequencing. Newly obtained sequences were compared with the National Center for Biotechnology Information database^1^ using the Basic Local Alignment Search Tool^2^ and related sequences were retrieved from the GenBank database.^3^ Selection of representative samples for use ClustalW software^4^ multiple sequence alignment. Phylogenetic trees were constructed using the Maximum Likelihood method with a Tamura 3-parameter model and bootstrapping of 1,000 replicates to calculate the evolutionary relationship using Molecular Evolutionary Genetics Analysis software (25).^5^

### 2.6 Risk factor analysis

Prism9 software (GraphPad Software, LLC, San Diego, CA, United States) was used for statistical analysis of tick-borne pathogen infections under different conditions. A Fisher score algorithm was used to select the optimal model. Univariate logistic regression was used to identify potential risk factors. A probability (*p*) value <0.05 was considered statistically significant. The odds ratio (OR) and 95% confidence interval (CI) were calculated to explore the correlation between the prevalence of pathogens and different factors. Ref represents the reference value for each set of data. Relevant data were expressed with reference to Zhao et al. (26).

## 3 Results

### 3.1 Tick species survey

**Figure 1:**
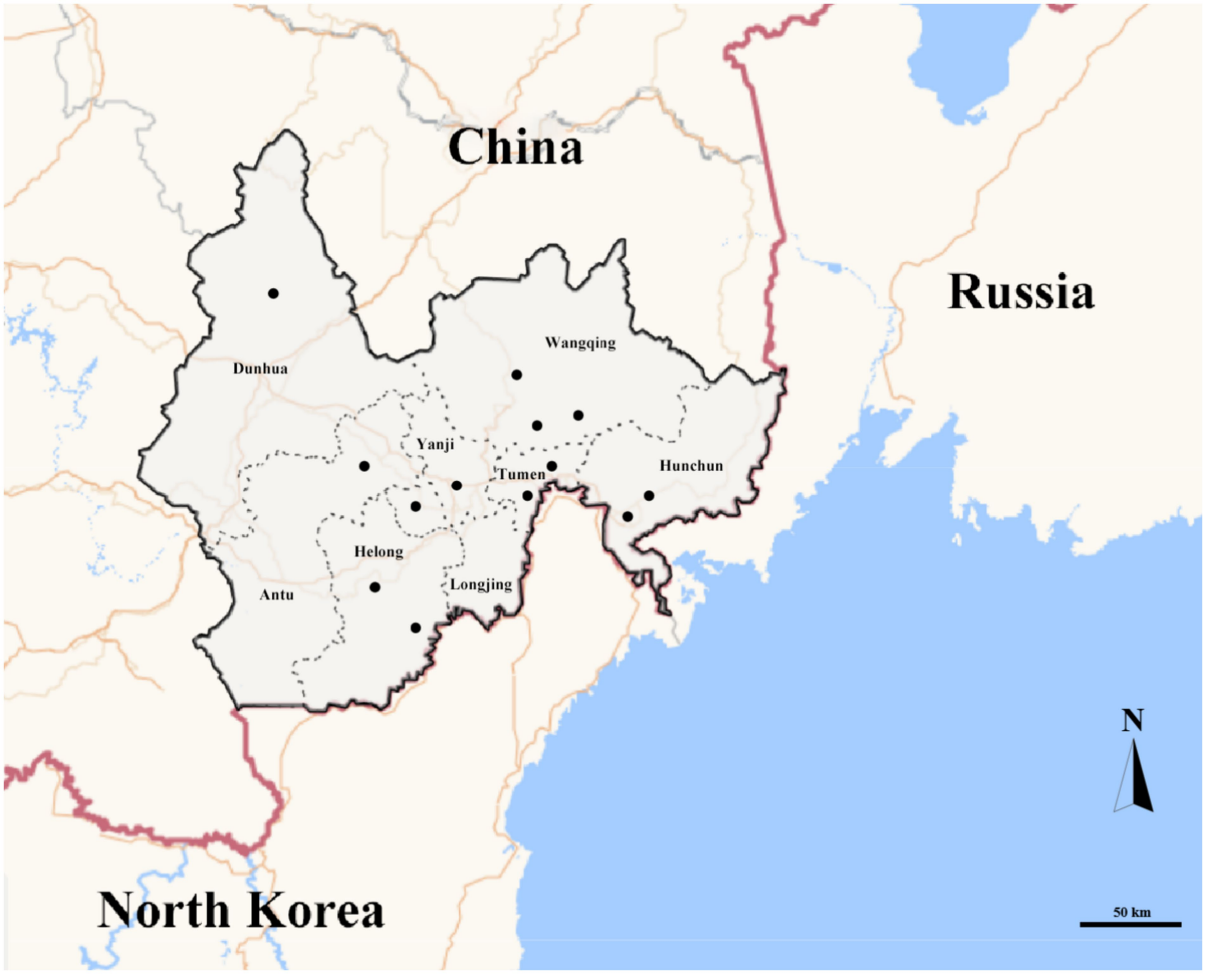
Map of in border areas of China, Russia and North Korea. Gray ranges represent sampling areas. Black dots represent sampling points.

In total, 1,187 ticks, were collected from 2020 to 2021 among eight counties of border areas of China (Hunchun, Yanji, Tumen, Longjing, Dunhua, Helong, Wangqing, and Antu), Russia, and North Korea (Figure 1). Of the 1,187 ticks, 632 were female, 376 were male, and 179 were nymphs. Regarding the environments, 343 ticks were collected in forests, 351 in grasslands, and 493 on animal surfaces. According to the identification results, there were three genera and five species of ticks in border areas of China, Russia, and North Korea, which included 471 *Haemaphysalis longicornis*, 184 *Haemaphysalis japonica*, 48 *Haemaphysalis concinna*, 301 *Ixodes persulcatus*, and 183 *Dermacentor silvarum* with proportions of 39.68, 15.50, 4.04, 25.36, and 15.42%, respectively. *Haemaphysalis* accounted for 59.22%, indicating that it was the dominant tick genus in border areas of China, Russia, and North Korea.

### 3.2 Detection rate of TBDs

The average infection rate of *Rickettsia, Bartonella, Anaplasma*, and *Babesia* was 48.78, 22.91, 35.05, and 5.14%, respectively. Among these pathogens, four were detected in Hunchun, Wangqing, Tumen, Dunhua and Longjing. *Anaplasma* was not detected in Yanji. *Rickettsia* and *Bartonella* were only detected in Helong and Antu. Four pathogens were detected in *H. longicornis*, *H. japonica*, and *I. persulcatus*, while *Rickettsia*, *Bartonella*, and *Anaplasma* were found in *D. silvarum*, and *Bartonella* and *Anaplasma* were identified in *H. concinna*. *Bartonella* was confirmed in all tick species. No *T. sinensis* and *T. orientalis* were detected in this survey (Table 3).

**Table 3:** The prevalence of tick-borne pathogens from tick samples in this study.

Location	Tick spp.	Detection of pathogen (No. positive)				
	Name	No. collected	*Rickettsia*	*Bartonella*	*Anaplasma*	*Babesia*
Hunchun	*H. longicornis*	471	227	39	206	41
	*H. japonica*	13	5	4	5	1
	*H. concinna*	8	0	7	1	0
	*I. persulcatus*	10	7	4	3	2
	*D. silvarum*	60	59	59	54	0
Wangqing	*H. japonica*	82	1	19	3	2
	*I. persulcatus*	151	108	10	34	5
	*D. silvarum*	13	13	10	10	0
Helong	*H. concinna*	40	0	15	0	0
	*I. persulcatus*	16	15	0	0	0
Tumen	*H. japonica*	26	0	1	2	2
	*D. silvarum*	43	15	9	8	0
Yanji	*H. japonica*	30	6	6	0	1
	*I. persulcatus*	29	4	19	0	2
Antu	*D. silvarum*	67	20	15	0	0
Dunhua	*I. persulcatus*	95	81	40	85	4
Longjing	*H. japonica*	33	18	15	5	1
Total		1,187	579	272	416	61

### 3.3 Molecular survey of pathogens in ticks

**Figure 2:**
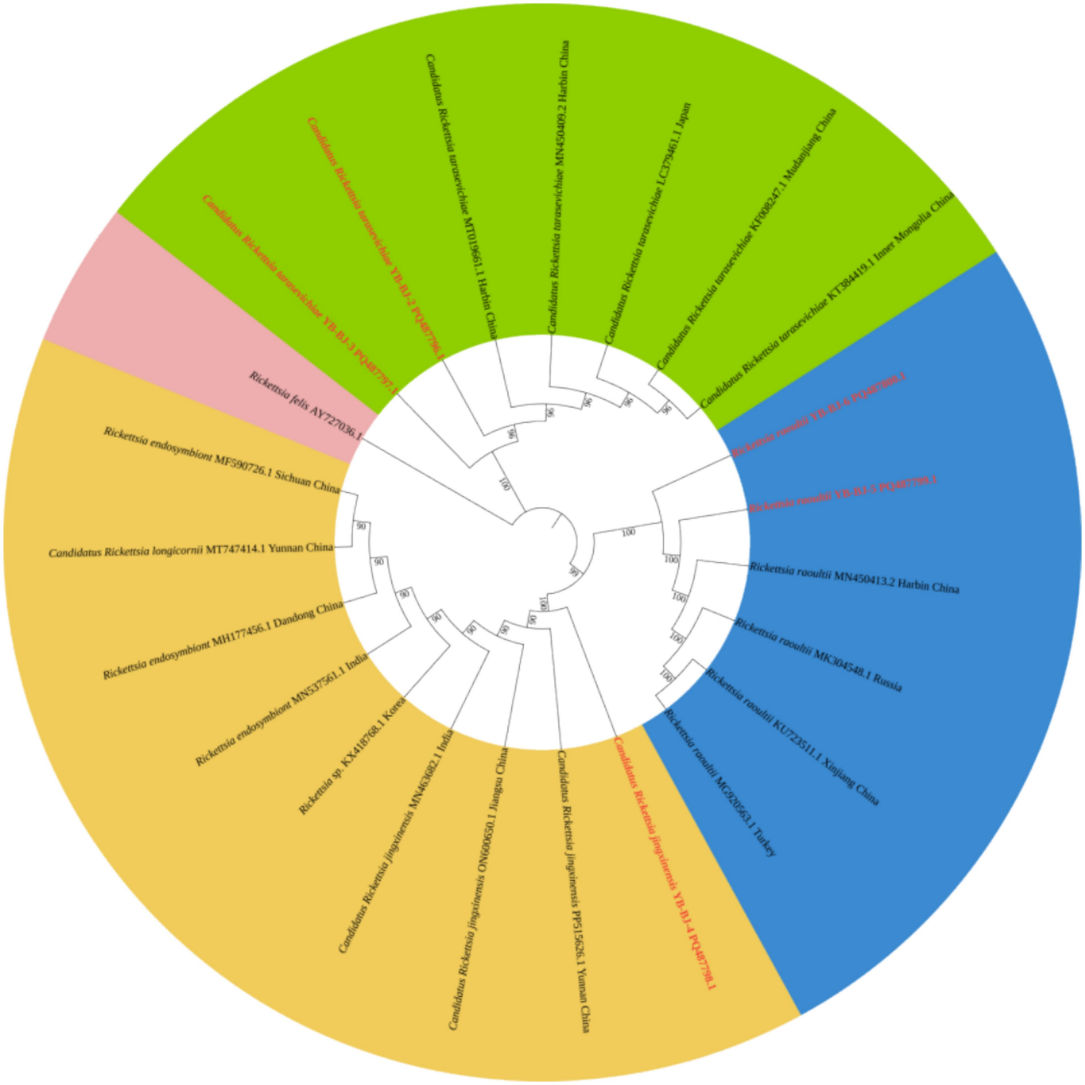
Phylogenetic analysis of *Rickettsia* based on *ompA* (530 bp). The phylogenetic trees were constructed by maximum-likelihood method and Tamura 3-parameter model with 1,000 bootstrap replications. The sequences obtained in this study are indicated with red color. The sequences of *Rickettsia felis* (AY727036) were included as outgroup.

**Figure 3:**
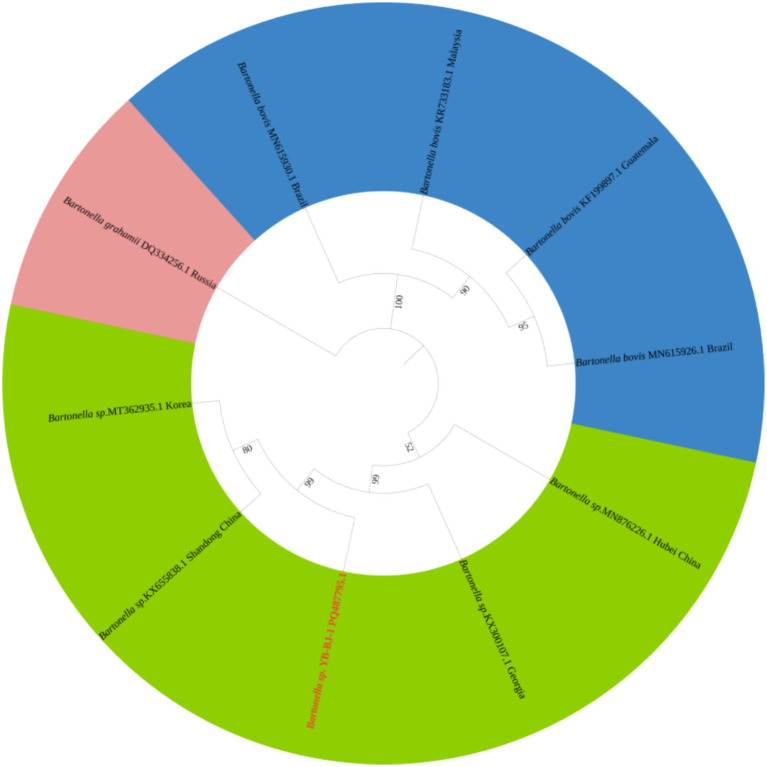
Phylogenetic analysis of *Bartonella* based on *gltA* (356 bp). The phylogenetic trees were constructed by maximum-likelihood method and Tamura 3-parameter model with 1,000 bootstrap replications. The sequences obtained in this study are indicated with red color. The sequences of *Bartonella grahamii* (DQ334256) were included as outgroup.

**Figure 4:**
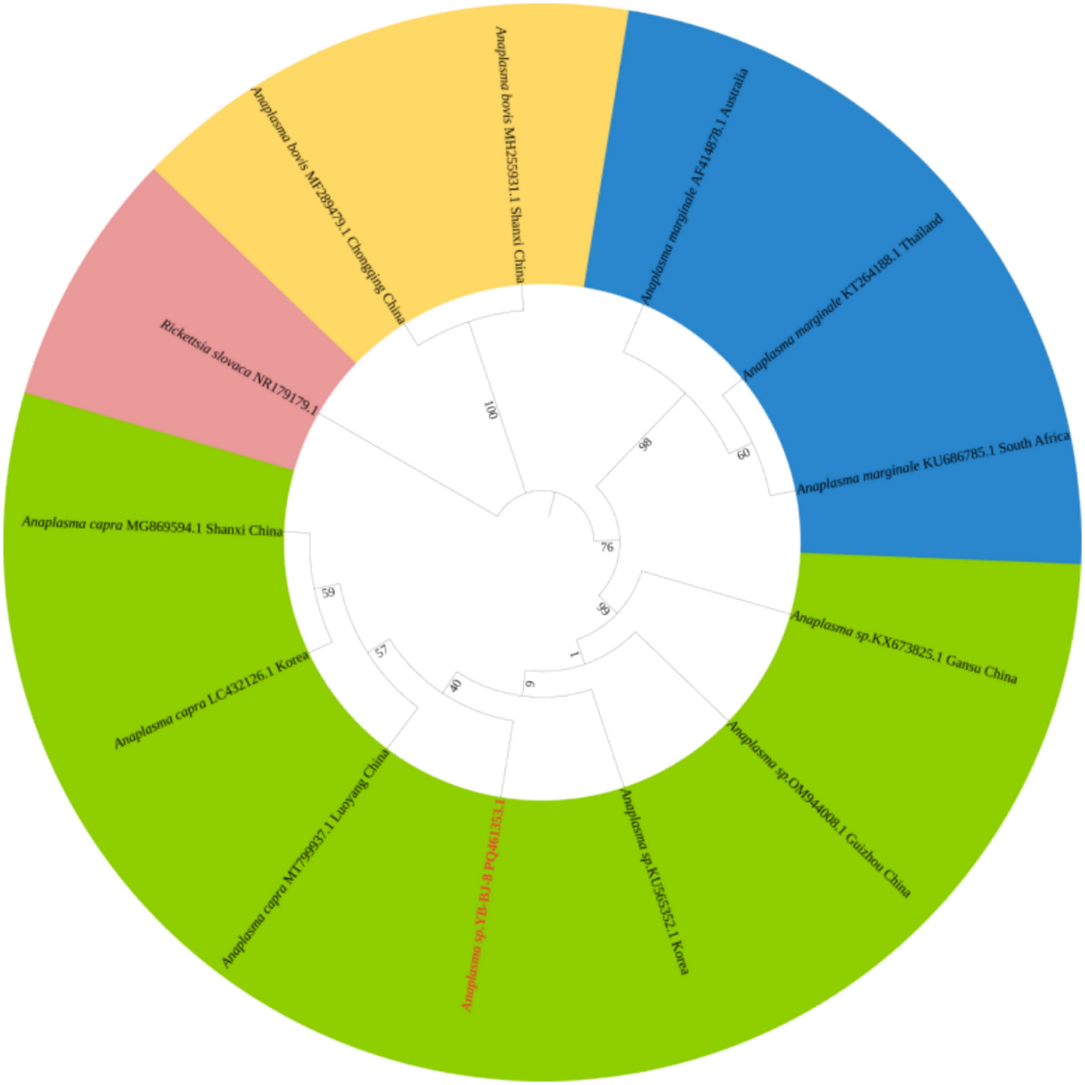
Phylogenetic analysis of *Anaplasma* based on *16SrRNA* (426 bp). The phylogenetic trees were constructed by maximum-likelihood method and Tamura 3-parameter model with 1,000 bootstrap replications. The sequences obtained in this study are indicated with red color. The sequences of *Rickettsia slovaca* (NR179179) were included as outgroup.

**Figure 5:**
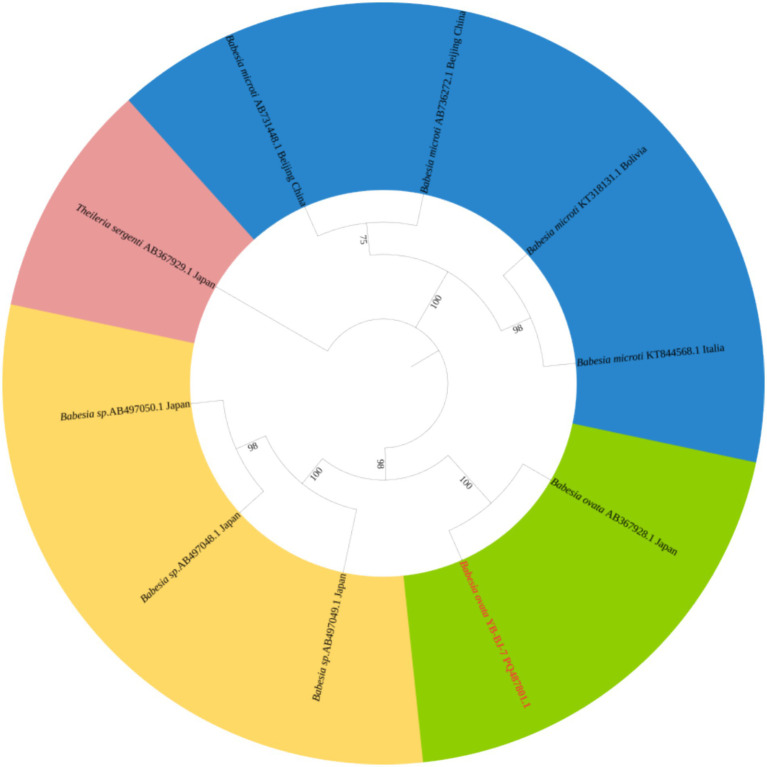
Phylogenetic analysis of *Babesia* based on *CCTeta* (1,008 bp). The phylogenetic trees were constructed by maximum-likelihood method and Tamura 3-parameter model with 1,000 bootstrap replications. The sequences obtained in this study are indicated with red color. The sequences of *Theileria sergenti* (AY727036) were included as outgroup.

The *Rickettsia* sequences were compared with known sequences in the GenBank database. Three genotypes of *Rickettsia* were detected in ticks in border areas of China, Russia, and North Korea: *Rickettsia raoultii*, *Candidatus rickettsia jingxinensis* and *Candidatus rickettsia tarasevichiae*. Among these, the YB-BJ-4 (PQ487798) strains were located in the same branch as isolates from India (MN537561), Sichuan province, China (MF590726), and Dandong, China (MH177456). The YB-BJ-5 (PQ487799) and YB-BJ-6 (PQ487800) strains were situated on the same branch as isolates from Siberia (MK304548), Turkey (MG920563), and Xinjiang, China (KU723511). The YB-BJ-2 (PQ487796) and YB-BJ-3 (PQ487797) strains were highly homologous and on the same branch with isolates from Harbin, China (MT019661), Mudanjiang, China (KF008247), and Hokkaido, Japan (LC379461) (Figure 2). The *Bartonella gltA* gene sequence obtained from this study (PQ487795) formed one clade with the Korea (MT362935) and Shandong province, China (KX655838) (Figure 3). Also, the *Anaplasma* sequence from this study (PQ461353) was compared with known *Anaplasma* sequences in the GenBank database. The isolates of *Anaplasma capra* from Luoyang, China (MT799937), Shanxi, China (MG869594), and Korea were located on the same branch and had the highest homology (Figure 4). Lastly, the *Babesia* sequence (PQ487801) was compared with known *Babesia ovata* sequences in the GenBank database. The isolate obtained in this study clustered with sequences from Japan (AB367928) with high homology (Figure 5).

### 3.4 Analysis of four pathogens under different factors

Our results suggest that regionally, ticks from four regions, Hunchun, Wangqing, Dunhua, and Longjing, may be more likely to carry *Rickettsia*; ticks from Yanji, Dunhua, and Longjing had a higher detection rate of *Bartonella*; ticks from two regions, Hunchun and Dunhua, were more likely to be infected with *Anaplasma*; and there was no significant difference in the distribution of *Babesia* across regions. When analyzed from the perspective of tick species*, H. longicornis, I. persulcatus,* and *D. silvarum* are more likely to carry *Rickettsia* and *Anaplasma*; *Bartonella* is more likely to be present in all four species of ticks except *H. longicornis*; and for the *Babesia*, *H. longicornis* is a likely potential vector. The sex of the tick is also an important factor in the prevalence of TBDs. Our study found that female ticks were more likely to carry *Rickettsia*, *Bartonella*, and *Babesia*; male ticks were more likely to carry *Anaplasma.* Finally, analyzing the collection environment we found that ticks from animal body surfaces are more likely to carry pathogens compared to the natural environment. In summary, region, tick species, sex, and collection environment may be potential risk factors for TBDs transmission (Tables 4–7).

**Table 4:** *Rickettsia* infection under different factors.

Factors	Category	No. of samples collected	No. of positive samples	Positive rate (%)	OR	95% CI	*p*- value
Region	Hunchun	562	298	53.02	5.53	2.75–11.14	<0.01
	Wangqing	246	122	49.59	4.82	2.34–9.95	<0.01
	Helong	56	15	26.79	1.79	0.73–4.42	0.20
	Tumen	69	15	21.74	1.36	0.56–3.31	0.50
	Yanji	59	10	16.95	Ref	–	–
	Antu	67	20	29.85	2.09	0.88–4.92	0.09
	Dunhua	95	81	85.26	28.35	11.69–68.75	<0.01
	Longjing	33	18	54.55	5.88	2.24–15.44	<0.01
Species	*H. longicornis*	471	227	48.20	4.78	3.10–7.35	<0.01
	*H. japonica*	184	30	16.30	Ref	–	–
	*H. concinna*	48	0	0	–	–	–
	*I. persulcatus*	301	215	71.43	12.83	8.07–20.42	<0.01
	*D. silvarum*	183	107	58.47	7.23	4.43–11.79	<0.01
Sex	Female	632	405	64.08	4.87	3.37–7.04	<0.01
	Male	376	126	33.51	1.38	0.93–2.04	0.11
	Nymphal	179	48	26.82	Ref	–	–
Environment	Forest	343	89	25.95	Ref	–	–
	Grass	351	188	53.56	3.29	2.39–4.53	<0.01
	Body surface	493	302	61.26	4.51	3.37–6.10	<0.01

Ref, reference; 95% CI, confdence interval; OR, odds ratio.

**Table 5:** *Bartonella* infection under different factors.

Factors	Category	No. of samples collected	No. of positive samples	Positive rate (%)	OR	95% CI	*p*- value
Region	Hunchun	562	113	20.11	1.49	0.74–2.99	0.27
	Wangqing	246	39	15.85	1.11	0.52–2.36	0.78
	Helong	56	15	26.79	2.16	0.88–5.28	0.09
	Tumen	69	10	14.49	Ref	–	–
	Yanji	59	25	42.37	4.34	1.86–10.11	<0.01
	Antu	67	15	22.39	1.70	0.70–4.12	0.24
	Dunhua	95	40	42.11	4.29	1.96–9.40	<0.01
	Longjing	33	15	45.45	4.92	1.89–12.82	<0.01
Species	*H. longicornis*	471	39	8.28	Ref	–	–
	*H. japonica*	184	45	24.46	3.59	2.24–5.74	<0.01
	*H. concinna*	48	22	45.83	9.37	4.87–18.06	<0.01
	*I. persulcatus*	301	73	24.25	3.55	2.33–5.40	<0.01
	*D. silvarum*	183	93	50.82	11.45	7.39–17.73	<0.01
Sex	Female	632	147	23.26	3.31	1.89–5.80	<0.01
	Male	376	110	29.26	4.52	2.55–8.02	<0.01
	nymphal	179	15	8.38	Ref	–	–
Environment	Forest	343	93	27.11	1.52	1.07–2.17	0.02
	Grass	351	69	19.66	Ref	–	–
	Body surface	493	110	22.31	1.17	0.84–1.65	0.35

Ref, reference; 95% CI, confdence interval; OR, odds ratio.

**Table 6:** *Anaplasma* infection under different factors.

Factors	Category	No. of samples collected	No. of positive samples	Positive rate (%)	OR	95% CI	*p*- value
Region	Hunchun	562	269	47.86	5.42	2.72–10.81	<0.01
	Wangqing	246	47	19.11	1.39	0.66–2.93	0.38
	Helong	56	0	0	–	–	–
	Tumen	69	10	14.49	Ref	–	–
	Yanji	59	0	0	–	–	–
	Antu	67	0	0	–	–	–
	Dunhua	95	85	89.47	50.15	19.64–128.1	<0.01
	Longjing	33	5	15.15	1.05	0.33–3.38	0.93
Species	*H. longicornis*	471	206	43.74	36.54	4.99–267.2	<0.01
	*H. japonica*	184	15	8.15	4.17	0.54–32.42	0.17
	*H. concinna*	48	1	2.08	Ref	–	–
	*I. persulcatus*	301	122	40.53	32.03	4.36–235.4	<0.01
	*D. silvarum*	183	72	39.34	30.49	4.11–226.0	<0.01
Sex	Female	632	214	33.86	1.53	1.05–2.22	0.03
	Male	376	157	41.76	2.14	1.44–3.17	<0.01
	Nymphal	179	45	25.14	Ref	–	–
Environment	Forest	343	77	22.45	Ref	–	–
	Grass	351	100	28.49	1.38	0.98–1.94	0.07
	Body surface	493	239	48.48	3.25	2.39–4.43	<0.01

Ref, reference; 95% CI, confdence interval; OR, odds ratio.

**Table 7:** *Babesia* infection under different factors.

Factors	Category	No. of samples collected	No. of positive samples	Positive rate (%)	OR	95%CI	*p*- value
Region	Hunchun	562	44	7.83	2.85	0.67–12.01	0.16
	Wangqing	246	7	2.85	0.98	0.20–4.84	0.98
	Helong	56	0	0	–	–	–
	Tumen	69	2	2.90	Ref	–	–
	Yanji	59	3	5.08	1.80	0.29–11.13	0.53
	Antu	67	0	0	–	–	–
	Dunhua	95	4	4.21	1.47	0.26–8.28	0.66
	Longjing	33	1	3.03	1.05	0.09–11.98	0.97
Species	*H. longicornis*	471	41	8.70	2.41	1.06–5.48	0.03
	*H. japonica*	184	7	3.80	Ref	–	–
	*H. concinna*	48	0	0	–	–	–
	*I. persulcatus*	301	13	4.32	1.14	0.45–2.92	0.78
	*D. silvarum*	183	0	0	–	–	–
Sex	Female	632	49	7.75	2.55	1.34–4.86	<0.01
	Male	376	12	3.19	Ref	–	–
	Nymphal	179	0	0	–	–	–
Environment	Forest	343	8	2.33	1.37	0.47–4.00	0.56
	Grass	351	6	1.71	Ref	–	–
	Body surface	493	47	9.53	6.06	2.56–14.34	<0.01

Ref, reference; 95% CI, confdence interval; OR, odds ratio.

## 4 Discussion

China has a vast border with many countries. The border between China, North Korea, and Russia lies in northeast China. Because the forest hydrology resources in this area are very rich, the endemic tick species have gradually diversified. Ticks and other vectors in the border zone can freely migrate to another country through a variety of routes, which may increase the risk of tick-borne diseases. In this study, 1,187 ticks collected from eight counties and cities in border areas of China, Russia, and North Korea were classified and analyzed. Among the five identified species, *Haemaphysalis* were the dominant tick species. *H. longicornis* was the most commonly detected species in this survey. *H. longicornis*, commonly known as the New Zealand cattle tick, is found mainly in East Asia and the Pacific region (27), and more recently in the United States and other countries in the Americas. *H. longicornis* can parasitize most warm-blooded animals, including humans and domestic animals, and spread a variety of pathogens (28), thus posing major hidden dangers to public safety and the animal husbandry industry.

In this study, three types of *Rickettsia* were detected. In fact, *R. raoultii* and *C. rickettsia tarasevichiae* have been endemic along the China-Russia border areas in recent years. Related studies have shown that *R. raoultii* and *C. rickettsia tarasevichiae* were detected in *I. persulcatus* and *D. silvarum* along the China-Russia border areas in 2014 (29, 30). This is almost consistent with the results of our survey. Notably, in previous studies, *C. rickettsia jingxinensis* have been reported mainly in Southwestern China and Korea (31, 32). The *C. rickettsia jingxinensis* detected in this survey were also consistent with the above areas in terms of their affinities. This suggests that there is potential for the spread of *C. rickettsia jingxinensis* to the border areas. *Bartonella* is widely prevalent around the world, and usually lice and fleas are considered to be the main vectors of *Bartonella* (33). Whether ticks are capable of transmitting *Bartonella* remains controversial. In 2022, some researchers from Portugal surveyed 268 ticks in Portugal and found that none of the ticks had infected *Bartonella* (34). However, a total of 272 *Bartonella* infections were detected in 1,187 ticks in our survey, indicating that ticks do have the ability to carry *Bartonella*. It remains to be investigated whether ticks can transmit *Bartonella* to their hosts through blood-sucking. *A. capra* is an emerging zoonotic tick-borne pathogen with a broad host range, including many mammals. In 2012, *A. capra* was detected in goats in China. Although current studies are not sufficient, domestic ruminants are considered the main host (35). Prior to this, *A. capra* was mainly prevalent in south-central China. *A. capra* have been reported to be detected in *H. longicornis* in Hubei Province, China, with a positivity rate of 1.32% (36). This is the first report of *A. capra* detected at the border areas of China, Russia, and North Korea. Gene sequences were in the same clade as the isolates from Luoyang and Shanxi. The positivity rate in this survey was significantly higher than in previous studies, a result that reminds us to pay close attention to the prevalence of *A. capra*. *B. ovata* is more frequently reported in Japan (37). The isolates from this investigation showed the highest homology with isolates from Japan. There are fewer reports on the epidemiology of *B. ovata*. It has been reported that 646 bovine bloods from various regions of China were positive for *B. ovata* at a rate of 1.5% (38). The positivity rate of *B. ovata* was also low in this survey. There are many reasons for this phenomenon, but of course scientific prevention and control is essential.

Our survey identified four potential risk factors that influence the prevalence of TBDs. Tick species are one of the most important factors in the prevalence of TBDs. Different species of ticks can carry different pathogens. It has been reported that *H. longicornis* can carry up to 44 pathogens. It’s one of the tick species that carries the highest number of pathogens (39). Our study found similar problems. Among the ticks we collected, *H. longicornis* was the most abundant and infected with four pathogens. The relationship between region and tick species is inextricably linked, and the distribution of ticks is significantly regional. In China, tick species are more abundant in the Northwestern and Southwestern regions, TBDs epidemics are also more severe. Although the samples were collected only in the border area of Northeast China, we can see from the results that some species of ticks were detected only in specific areas. Interestingly, sex and collection environment were also found to be risk factors for TBDs, and although the exact reasons for this are unclear, this phenomenon deserves to be studied in depth.

## 5 Conclusion

*Haemaphysalis* are the dominant tick genus in border areas of China, Russia, and North Korea. Four pathogens (*Rickettsia*, *Bartonella*, *Anaplasma,* and *Babesia*) were detected in the tick species collected in this study. Based on our results, scientific exclusion of potential risk factors may provide a new idea for controlling the spread of tick-borne diseases. These findings provide epidemiological data to support the prevention and control of ticks and tick-borne diseases in the border region of China, Russia, and North Korea.

## Data Availability

The datasets presented in this study can be found in online repositories. The names of the repository/repositories and accession number(s) can be found in the article/Supplementary material.

## Footnotes

^1^ https://www.ncbi.nlm.nih.gov/

^2^ https://blast.ncbi.nlm.nih.gov/Blast.cgi

^3^ https://www.ncbi.nlm.nih.gov/genbank/

^4^ http://www.clustal.org/

^5^v.11.0; https://www.megasoftware.net/

## References

[1] Nava S Guglielmone AA Mangold AJ. An overview of systematics and evolution of ticks. Front Biosci (Landmark Ed). (2009) 14:2857–77. doi: 10.2741/3418, PMID: 19273240

[2] Horak IG Camicas JL Keirans JE. The Argasidae, Ixodidae and Nuttalliellidae (Acari: Ixodida): a world list of valid tick names. Exp Appl Acarol. (2002) 28:27–54. doi: 10.1023/a:1025381712339, PMID: 14570115

[3] Lu X Lin XD Wang JB Qin XC Tian JH Guo WP. Molecular survey of hard ticks in endemic areas of tick-borne diseases in China. Ticks Tick Borne Dis. (2013) 4:288–96. doi: 10.1016/j.ttbdis.2013.01.003 23538111

[4] Song R Ma Y Hu Z Li Y Li M Wu L. MaxEnt modeling of *Dermacentor marginatus* (Acari: Ixodidae) distribution in Xinjiang, China. J Med Entomol. (2020) 57:1659–67. doi: 10.1093/jme/tjaa063, PMID: 32359141

[5] Brophy M Walker KR Adamson JE Ravenscraft A. Tropical and temperate lineages of *Rhipicephalus sanguineus* s.l. ticks (Acari: Ixodidae) host different strains of Coxiella-like endosymbionts. J Med Entomol. (2022) 59:2022–9. doi: 10.1093/jme/tjac132, PMID: 36124671

[6] Chen Z Liu J. A review of argasid ticks and associated pathogens of China. Front Vet Sci. (2022) 9:865664. doi: 10.3389/fvets.2022.865664 35958318 PMC9361067

[7] Mansfield KL Schilling M Sanders C Holding M Johnson N. Arthropod-borne viruses of human and animal importance: overwintering in temperate regions of Europe during an era of climate change. Microorganisms. (2024) 12:1307. doi: 10.3390/microorganisms12071307, PMID: 39065076 PMC11278640

[8] McCoy KD Toty C Dupraz M Tornos J Gamble A Garnier R. Climate change in the Arctic: testing the poleward expansion of ticks and tick-borne diseases. Glob Chang Biol. (2023) 29:1729–40. doi: 10.1111/gcb.16617, PMID: 36700347

[9] Chisu V Giua L Bianco P Masala G Sechi S Cocco R. Molecular survey of Hepatozoon canis infection in domestic dogs from Sardinia, Italy. Vet Sci. (2023) 10:640. doi: 10.3390/vetsci10110640, PMID: 37999463 PMC10674782

[10] Barker SC Walker AR. Ticks of Australia. The species that infest domestic animals and humans. Zootaxa. (2014) 3816:1–144. doi: 10.11646/zootaxa.3816.1.1, PMID: 24943801

[11] Rodriguez Y Rojas M Gershwin ME Anaya JM. Tick-borne diseases and autoimmunity: a comprehensive review. J Autoimmun. (2018) 88:21–42. doi: 10.1016/j.jaut.2017.11.007, PMID: 29183642

[12] Kim HK. Rickettsia-host-tick interactions: knowledge advances and gaps. Infect Immun. (2022) 90:e0062121. doi: 10.1128/iai.00621-21, PMID: 35993770 PMC9476906

[13] Gillespie JJ Williams K Shukla M Snyder EE Nordberg EK Ceraul SM. Rickettsia phylogenomics: unwinding the intricacies of obligate intracellular life. PLoS One. (2008) 3:e2018. doi: 10.1371/journal.pone.0002018, PMID: 19194535 PMC2635572

[14] Biswal M Krishnamoorthi S Bisht K Sehgal A Kaur J Sharma N. Rickettsial diseases: not uncommon causes of acute febrile illness in India. Trop med. Infect Dis. (2020) 5:59. doi: 10.3390/tropicalmed5020059, PMID: 32326477 PMC7344935

[15] Woldehiwet Z. The natural history of *Anaplasma phagocytophilum*. Vet Parasitol. (2010) 167:108–22. doi: 10.1016/j.vetpar.2009.09.013, PMID: 19811878

[16] Kocan KM de la Fuente J Blouin EF Coetzee JF Ewing SA. The natural history of *Anaplasma marginale*. Vet Parasitol. (2010) 167:95–107. doi: 10.1016/j.vetpar.2009.09.012, PMID: 19811876

[17] Kolo A. Anaplasma species in Africa-a century of discovery: a review on molecular epidemiology, genetic diversity, and control. Pathogens. (2023) 12:702. doi: 10.3390/pathogens12050702, PMID: 37242372 PMC10222256

[18] Deng H Le Rhun D Buffet JP Cotte V Read A Birtles RJ. Strategies of exploitation of mammalian reservoirs by Bartonella species. Vet Res. (2012) 43:15. doi: 10.1186/1297-9716-43-15, PMID: 22369683 PMC3430587

[19] Nelson CA Saha S Mead PS. Cat-scratch disease in the United States, 2005-2013. Emerg Infect Dis. (2016) 22:1741–6. doi: 10.3201/eid2210.160115, PMID: 27648778 PMC5038427

[20] Vannier EG Diuk-Wasser MA Ben Mamoun C Krause PJ. Babesiosis. Infect Dis Clin N Am. (2015) 29:357–70. doi: 10.1016/j.idc.2015.02.008, PMID: 25999229 PMC4458703

[21] Bal MS Mahajan V Filia G Kaur P Singh A. Diagnosis and management of bovine babesiosis outbreaks in cattle in Punjab state. Vet World. (2016) 9:1370–4. doi: 10.14202/vetworld.2016.1370-1374, PMID: 28096607 PMC5234049

[22] Wikel SK. Tick-host-pathogen systems immunobiology: an interactive trio. Front Biosci (Landmark Ed). (2018) 23:265–83. doi: 10.2741/4590, PMID: 28930546

[23] Li J Zhang S Liang W Zhao S Wang Z Li H. Survey of tick species and molecular detection of selected tick-borne pathogens in Yanbian, China. Parasite. (2022) 29:38. doi: 10.1051/parasite/2022039, PMID: 35861542 PMC9302104

[24] Keirans JE Litwak TR. Pictorial key to the adults of hard ticks, family Ixodidae (Ixodida: Ixodoidea), east of the Mississippi River. J Med Entomol. (1989) 26:435–48. doi: 10.1093/jmedent/26.5.435, PMID: 2795615

[25] Alkathiri B Lee S Ahn K Cho YS Youn SY Seo K. 16S rRNA metabarcoding for the identification of tick-borne bacteria in ticks in the Republic of Korea. Sci Rep. (2024) 14:19708. doi: 10.1038/s41598-024-70815-7, PMID: 39181959 PMC11344767

[26] Zhao S Wang H Zhang S Xie S Li H Zhang X. First report of genetic diversity and risk factor analysis of equine piroplasm infection in equids in Jilin, China. Parasit Vectors. (2020) 13:459. doi: 10.1186/s13071-020-04338-1, PMID: 32907616 PMC7479743

[27] Gunton AL Jenkins SJ. Chemical softness in aromatic adsorption: benzene, nitrobenzene and anisole on Pt111. J Phys Chem A. (2024) 128:6296–304. doi: 10.1021/acs.jpca.4c02214, PMID: 39037904 PMC11299172

[28] Beard CB Occi J Bonilla DL Egizi AM Fonseca DM Mertins JW. Multistate infestation with the exotic disease-vector tick *Haemaphysalis longicornis* - United States, august 2017-September 2018. MMWR Morb Mortal Wkly Rep. (2018) 67:1310–3. doi: 10.15585/mmwr.mm6747a3, PMID: 30496158 PMC6276380

[29] Wen J Jiao D Wang JH Yao DH Liu ZX Zhao G. *Rickettsia raoultii*, the predominant Rickettsia found in *Dermacentor silvarum* ticks in China-Russia border areas. Exp Appl Acarol. (2014) 63:579–85. doi: 10.1007/s10493-014-9792-0, PMID: 24699771

[30] Yi S Hongrong J Wuchun C Weiming F Wendong J Xin W. Prevalence of Candidatus Rickettsia tarasevichiae-like Bacteria in Ixodid ticks at 13 sites on the Chinese-Russian border. J Med Entomol. (2014) 51:1304–7. doi: 10.1603/ME13189, PMID: 26309321

[31] Park HJ Kim J Choi YJ Kim HC Klein TA Chong ST. Tick-borne rickettsiae in Midwestern region of Republic of Korea. Acta Trop. (2021) 215:105794. doi: 10.1016/j.actatropica.2020.105794, PMID: 33310079

[32] Lu M Meng C Zhang B Wang X Tian J Tang G. Prevalence of spotted fever group Rickettsia and Candidatus Lariskella in multiple tick species from Guizhou Province, China. Biomol Ther. (2022) 12:701. doi: 10.3390/biom12111701, PMID: 36421715 PMC9688252

[33] Spitalska E Minichova L Hamsikova Z Stanko M Kazimirova M. *Bartonella*, *Rickettsia*, *Babesia*, and Hepatozoon species in fleas (Siphonaptera) infesting small mammals of Slovakia (Central Europe). Pathogens. (2022) 11:886. doi: 10.3390/pathogens11080886, PMID: 36015007 PMC9413308

[34] Torrejon E Sanches GS Moerbeck L Santos L Andre MR Domingos A. Molecular survey of Bartonella species in stray cats and dogs, humans, and questing ticks from Portugal. Pathogens. (2022) 11:749. doi: 10.3390/pathogens11070749, PMID: 35889995 PMC9323395

[35] Altay K Erol U Sahin OF. Anaplasma capra: a new emerging tick-borne zoonotic pathogen. Vet Res Commun. (2024) 48:1329–40. doi: 10.1007/s11259-024-10337-9, PMID: 38424380 PMC11147849

[36] Tang J Xu J Liu XH Lv FZ Yao QJ Zhou XF. Prevalence and genetic diversity of Anaplasma and Ehrlichia in ticks and domesticated animals in Suizhou County, Hubei Province, China. Sci Rep. (2024) 14:12621. doi: 10.1038/s41598-024-63267-6, PMID: 38824201 PMC11144266

[37] Sivakumar T Tattiyapong M Okubo K Suganuma K Hayashida K Igarashi I. PCR detection of Babesia ovata from questing ticks in Japan. Ticks Tick Borne Dis. (2014) 5:305–10. doi: 10.1016/j.ttbdis.2013.12.006, PMID: 24572609

[38] Niu Q Liu Z Yu P Yang J Abdallah MO Guan G. Genetic characterization and molecular survey of Babesia bovis, Babesia bigemina and Babesia ovata in cattle, dairy cattle and yaks in China. Parasit Vectors. (2015) 8:518. doi: 10.1186/s13071-015-1110-0, PMID: 26452623 PMC4600270

[39] Zhao GP Wang YX Fan ZW Ji Y Liu MJ Zhang WH. Mapping ticks and tick-borne pathogens in China. Nat Commun. (2021) 12:1075. doi: 10.1038/s41467-021-21375-1, PMID: 33597544 PMC7889899

[40] Yang J Liu Z Niu Q Liu J Xie J Chen Q. Evaluation of different nested PCRs for detection of *Anaplasma phagocytophilum* in ruminants and ticks. BMC Vet Res. (2016) 12:35. doi: 10.1186/s12917-016-0663-2, PMID: 26911835 PMC4765105

[41] Jia N Jiang JF Huo QB Jiang BG Cao WC. *Rickettsia sibirica* subspecies *sibirica* BJ-90 as a cause of human disease. N Engl J Med. (2013) 369:1176–8. doi: 10.1056/NEJMc1303625, PMID: 24047079

[42] Qin XR Han HJ Han FJ Zhao FM Zhang ZT Xue ZF. Rickettsia japonica and novel Rickettsia species in ticks, China. Emerg Infect Dis. (2019) 25:992–5. doi: 10.3201/eid2505.171745, PMID: 31002060 PMC6478201

[43] Liu A Guan G Liu Z Liu J Leblanc N Li Y. Detecting and differentiating Theileria sergenti and Theileria sinensis in cattle and yaks by PCR based on major piroplasm surface protein (MPSP). Exp Parasitol. (2010) 126:476–81. doi: 10.1016/j.exppara.2010.05.024, PMID: 20685208

[44] Ota N Mizuno D Kuboki N Igarashi I Nakamura Y Yamashina H. Epidemiological survey of theileria orientalis infection in grazing cattle in the eastern part of Hokkaido, Japan. Nippon Juigaku Zasshi. (2009) 71:937–44. doi: 10.1292/jvms.71.937, PMID: 19652482

[45] Vial L Durand P Arnathau C Halos L Diatta G Trape JF. Molecular divergences of the *Ornithodoros sonrai* soft tick species, a vector of human relapsing fever in West Africa. Microbes Infect. (2006) 8:2605–11. doi: 10.1016/j.micinf.2006.07.012, PMID: 16962358

